# Assessment of microbial communities from cold mine environments and subsequent enrichment, isolation and characterization of putative antimony- or copper-metabolizing microorganisms

**DOI:** 10.3389/fmicb.2024.1386120

**Published:** 2024-05-24

**Authors:** Francisca Prieto-Fernández, Stefan Lambert, Katharina Kujala

**Affiliations:** Water, Energy and Environmental, Engineering Research Unit, University of Oulu, Oulu, Finland

**Keywords:** metal, metalloid, cold, copper, antimony, enrichment, isolation, bioremediation

## Abstract

Mining activities, even in arctic regions, create waste materials releasing metals and metalloids, which have an impact on the microorganisms inhabiting their surroundings. Some species can persist in these areas through tolerance to meta(loid)s via, e.g., metabolic transformations. Due to the interaction between microorganisms and meta(loid)s, interest in the investigation of microbial communities and their possible applications (like bioremediation or biomining) has increased. The main goal of the present study was to identify, isolate, and characterize microorganisms, from subarctic mine sites, tolerant to the metalloid antimony (Sb) and the metal copper (Cu). During both summer and winter, samples were collected from Finnish mine sites (site A and B, tailings, and site C, a water-treatment peatland) and environmental parameters were assessed. Microorganisms tolerant to Sb and Cu were successfully enriched under low temperatures (4°C), creating conditions that promoted the growth of aerobic and fermenting metal(loid) tolerating or anaerobic metal(loid) respiring organism. Microbial communities from the environment and Sb/Cu-enriched microorganisms were studied via 16S rRNA amplicon sequencing. Site C had the highest number of taxa and for all sites, an expected loss of biodiversity occurred when enriching the samples, with genera like *Prauserella, Pseudomonas* or *Clostridium* increasing their relative abundances and others like *Corynebacterium* or *Kocuria* reducing in relative abundance. From enrichments, 65 putative Sb- and Cu-metabolizing microorganisms were isolated, showing growth at 0.1 mM to 10 mM concentrations and 0°C to 40°C temperatures. 16S rRNA gene sequencing of the isolates indicated that most of the putative anaerobically Sb-respiring tolerators were related to the genus *Clostridium*. This study represents the first isolation, to our knowledge, of putative Sb-metabolizing cold-tolerant microorganisms and contributes to the understanding of metal (loid)-tolerant microbial communities in Arctic mine sites.

## Graphical abstract

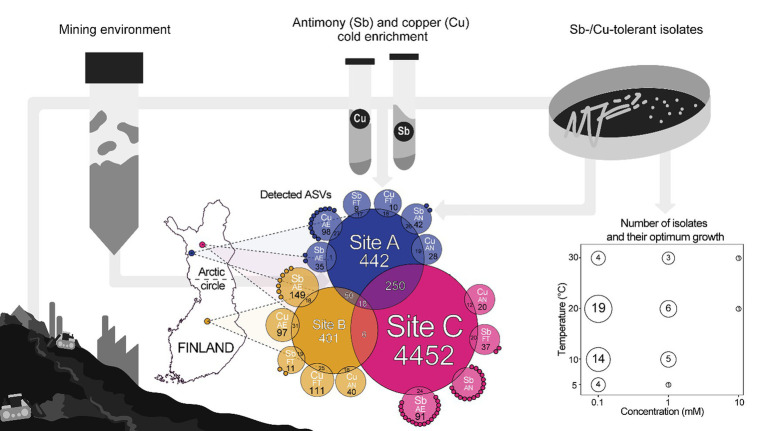

## Introduction

Mining activities generate substantial amounts of waste materials and those can result in the release of metals and metalloids to ecosystems. Even in (sub-)arctic sites such as Svalbard, Alaska or Finland, metal(loid)s are present as a consequence of the mining industry (Perryman et al., 2020; Rudnicka-Kępa and Zaborska, 2021; Haddaway et al., 2022). Although metal(loid)s in waste materials might be potentially valuable resources (Falagán et al., 2017), when those pollutants end up in nature, they can negatively impact plants, animals or microbial communities inhabiting those sites (Ledin, 1996). Some microorganisms with the ability of tolerate metal(loid)s can colonize and inhabit highly contaminated places by using strategies such as metabolic pathways or resistance mechanisms (Newsome and Falagán, 2021). Because of the ability of certain microorganisms to transform metal(loid)s, there is an interest in studying microbial communities from mining contaminated sites and their potential industrial applications (Levett et al., 2021; Staicu and Stolz, 2021). However, there are still many gaps to fill in the knowledge on microorganisms from polluted places and their interaction with elements in nature, especially in extreme places, like arctic regions.

One example of mining-impacted sites are mine tailings, the waste or the material left over after the valuable component has been extracted (Roche et al., 2017). Because of the chemical properties of mine tailings, their environmental impact depends on their potential to release metal(loids)s into natural ecosystems (Jiang et al., 2021; Cacciuttolo et al., 2023). Another example of a place highly impacted by mining activities are the systems utilized for the purification of mining discharges, like peatlands used for passive mine water treatment purposes (Palmer et al., 2015). Due to the characteristics of peatlands, e.g., high water tables or large effective surface area, different metal(loids)s can accumulate in these environments through different pathways such as atmospheric deposition or contaminated groundwater (Steinnes and Friedland, 2006; Palmer et al., 2015; Eberle et al., 2021). Both tailings and mine water-treatment peatlands represent an ecological challenge due to the presence of toxic elements, but it could also serve as a potential sources of valuable minerals which, if not reutilized, result in economic loss (Falagán et al., 2017; Sethurajan et al., 2018; Aznar-Sánchez et al., 2019).

Microorganisms can be used for several industrial purposes. For instance, microorganisms can be used in bioremediation, a method to biologically remove contaminants from ecosystems (Dixit et al., 2015; Malla et al., 2018). This technique has been previously studied and applied in mining-affected environments in an attempt to return the ecosystems to their natural state (Newsome and Falagán, 2021; Bala et al., 2022; Riseh et al., 2023; Sánchez-Castro et al., 2023). Microorganisms can also be used for biomining processes, the extraction of metals of interest from their ores aided through biological processes such as bioleaching or bioaccumulation (Jerez, 2017). Microbial contribution in the obtention of metal(loid)s has been demonstrated, including recovery from mine waste materials (Gao et al., 2021; Chen W. et al., 2022). In the mine areas we had access to in our study, antimony (Sb) and copper (Cu) are of particular interest. Antimony is an element that has been listed as a priority pollutant internationally (United States Environmental Protection Agency, 1997; European Parliament, Council of the European Union, 2020) and a critical raw material for the EU (Grohol et al., 2023). The awareness towards Sb has grown in the past years because of its detrimental effects to humans and the environment (Bolan et al., 2022; Periferakis et al., 2022). Industrial operations, such as mining, contribute to the release of Sb to surrounding native areas (Qi et al., 2022). However, Sb is a non-renewable and valuable element whose importance is expected to continue increasing because of its role in the energy transition (Li et al., 2022). Another example of precious raw material is Cu. Nowadays, one of the concerns in the mining industry is that usually small traces of Cu, which were not extracted in the first place, are left in the material after the extractive operations (Loredo Portales et al., 2015; Falagán et al., 2017). Thus, efforts are needed to remove Sb, from mine sites to reduce contamination of natural ecosystems and to develop alternative supplies of Sb and Cu, like recovering less available and less concentrated Sb and Cu from mine waste (Falagán et al., 2017; Sun et al., 2018; Grohol et al., 2023).

Different approaches have been described for Sb bioremediation like antimonite [Sb(III)] oxidation which decreases the Sb toxicity (Hamamura et al., 2013; Li et al., 2019) or antimonate [Sb(V)] reduction and subsequent precipitation (Wang et al., 2013, 2018; Zhu et al., 2018). However, regardless of its promising significance, little is known about microorganisms that participate in Sb(V) reduction when comparing to, e.g., Sb(III) oxidation (Li et al., 2019). In fact, only a few Sb(V) reducing microbes have been isolated (Chen Y. et al., 2022), with the first organisms described only a decade ago (Kulp et al., 2014). The relationship between Sb and microorganisms has been studied for other applications as well. For example, it has been proven that microorganisms can contribute in the recovery of Sb from mining ores through bioleaching (Hafeez et al., 2017; Aghazadeh et al., 2023). However, Cu is the metal which has been most extensively researched for biomining. For instance, Cu biomining systems have been applied internationally (Jerez, 2017; Yin et al., 2018; Vera et al., 2022). In addition, Cu-recovery from waste materials has also been developed (Falagán et al., 2017; Chen W. et al., 2022). Several species isolated from mine sites have been reported for their bioleaching activities of Cu (Kumar and Nagendran, 2007; Govarthanan et al., 2014), and most of them are known to be able to withstand very high Cu concentrations (Dopson et al., 2003; Orell et al., 2010). Nonetheless, despite the mentioned studies assessing Sb and Cu-tolerant microorganisms, only a few species have been isolated from cold environments or reported growth at low temperatures. From our knowledge, no psychrophilic microorganism [with an optimal growth temperature below 15°C (Morita, 1975)] or psychrotolerant microorganism [capable of growing at temperatures up to 0°C but with optimum growth temperature at 20°C or higher (Morita, 1975)] with Sb-resistance has been isolated. In addition, only a small number of studies have focused on characterizing psychrotolerant Cu-resistant microorganisms (Barahona et al., 2014; Muñoz-Villagrán et al., 2022). Therefore, with the rise of mining operations in Northern regions such as Finland, one of the top countries in the world in terms of mineral exploration (Tuusjärvi et al., 2014; Mejía and Aliakbari, 2023), research should focus on isolating, identifying and characterizing psychrophilic and psychrotolerant Sb and Cu-metabolizing microorganisms, as they must withstand the challenging temperatures of the mining industry in the Arctic as well as metal(loid) concentration to be viable for *in-situ* systems applications.

The goal of this study was to assess the diversity of microorganisms from mining-impacted sites in subarctic regions and isolate microorganisms that have potential for bioremediation and biomining applications. The aims of our study were to (1) compare microbial communities from different mining environments and the environmental parameters shaping the microbial community structure, (2) enrich and isolate microorganisms from environmental samples which have tolerance for our metals of interest, Sb and Cu, under cold conditions (4°C), (3) characterize the isolated microorganisms, and (4) asses their potential for further biotechnology applications. To address these aims, we obtained samples from mining contaminated sites in Northern and Central Finland in different seasons and studied their microbial communities via 16S rRNA gene sequencing and the influence of environmental parameters on them. Antimony and Cu tolerant microorganisms were selectively enriched in cold, *in-situ* relevant temperatures and subsequently isolated and characterized. Our study adds to the understanding of cold-adapted microorganisms from mining impacted environments and their potential use in biotechnology.

## Materials and methods

### Sampling and physicochemical analyses of environmental samples

**Figure 1:**
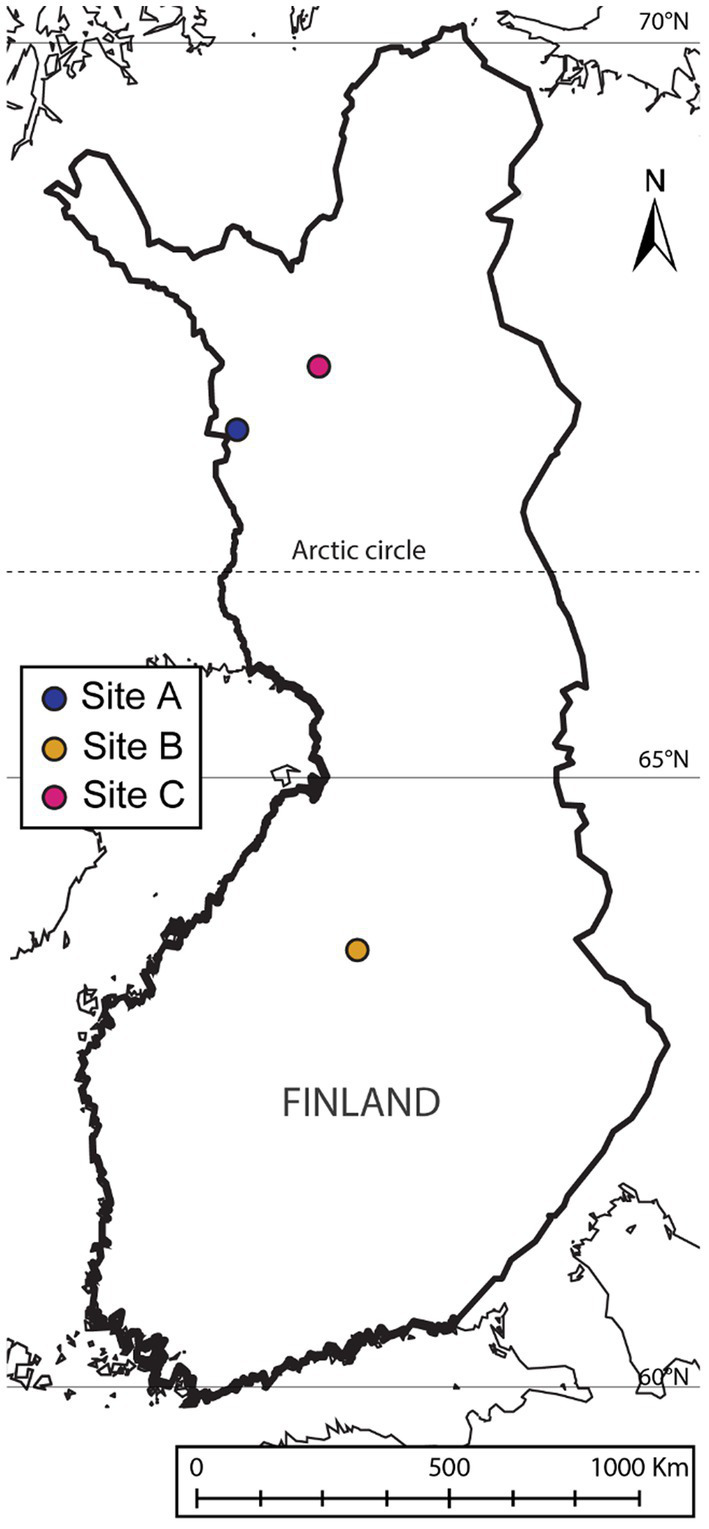
Sampling locations. Figure made with ArcGisPro [using basemap “Light Gray Canvas Map” (source: Esri, DeLorme, HERE, MapmyIndia) and layers “World Latitude and Longitude Grids” and “World GeoReference Lines” (Esri Data and Maps)].

Samples were obtained from three Finnish mine areas: one tailing from an abandoned mine (site A), one tailing from an active mine (site B) and one peatland used for mine water treatment in an active mine (site C) (Figure 1). Sites were selected based on the chemical element composition, with special emphasis on the Sb and Cu concentrations.

Summer sampling of site B was conducted at the end of August (24.08.2021) and sites A and C beginning of September (01.09.2021), while winter sampling for all sites took place in February (16.02.2022 for A, 08.02.2022 for B, and 15.02.2022 for C). Environmental samples were collected at different depths within the soil profile by excavating holes using a shovel, with one hole in site A and C and two holes in site B. During winter sampling, an ice drill was used to penetrate the superficial frozen layer. All samples were transported for no more than three hours in a cold box at 4°C until they were frozen and kept at −20°C until further analysis. Three subsamples were taken for all sites/depths/sampling occasions: one of the subsamples was used to determine pH, one subsample was used to determine elemental composition at a commercial laboratory (Eurofins Ahma Oy) and one subsample was used for DNA and RNA extraction and, in the case of the summer samples, as inoculant for the enrichment experiments. The week following each sampling total elemental concentrations of Sb, Cu, arsenic (As), sulfur (S) and iron (Fe) were determined after acidic microwave digestion by inductively coupled plasma mass spectrometry (ICP-MS) in a commercial laboratory (Eurofins Ahma Oy). The pH was measured in a 1:2 (w/v) aqueous solution using a pH meter (InoLab^®^ Multi 9,420 IDS).

### Enrichment and isolation of Sb- and Cu- tolerant microorganisms

Microbes from the environmental samples collected in summer were cultivated in three different growth media to selectively enrich Sb and Cu utilizing microorganisms: aerobic Sb or Cu tolerators, fermenting Sb or Cu tolerators and anaerobically Sb or Cu respiring microorganisms. Media were prepared using an artificial porewater solution containing essential minerals, trace elements, and vitamins [modified from Balch et al. (1979) and Kuhner et al. (1996)]. Media for aerobic and fermenting Sb and Cu tolerators was supplemented with 1:10 diluted nutrient broth as carbon source and media for anaerobic Sb and Cu respires with 1:100 diluted nutrient broth as well as sodium acetate and sodium lactate (5 mM each) as carbon source. The pH of all growth media was adjusted to 6.5. Anoxic media for fermenting tolerators and anaerobic respirers were prepared using modified Hungate techniques (Wüst et al., 2009). After autoclaving the media, 0.2 μm-filtered stock solutions of Sb or Cu were added to reach final concentrations of 1 mM. Antimonite (supplemented as Sb_2_O_3_) was used for the cultures of aerobic antimony-tolerant microorganisms, while antimonate (supplemented as H_6_KO_6_Sb) was added as a potential electron acceptor for fermenting antimony-tolerators and anaerobical antimonate respirers. Copper (supplemented as CuSO_4_) was utilized for the medium of aerobic and fermenting copper-tolerators as well as anaerobic copper- or sulfate-reducer, which would potentially use copper [as Cu(II)] or sulfate (SO4^2−^) as an electron acceptor. To prepare the sample for inoculation, 1 g of content from each of the environmental samples were placed in test tubes with 9 mL of distilled water (1:10 dilution) and shaken for ten minutes.

After the media were prepared, two approaches were used: enrichment culturing and dilution-to extinction protocols. For the enrichment culturing, 0.5 mL of each of the environmental solutions were transferred to 15 mL media contained in either 50 mL falcon tubes (aerobic) or sealed glass tubes (anoxic). For the dilution-to-extinction approach, 96-well plates were inoculated with different dilutions (10^−3^, 10^−4^ and 10^−5^) of the environmental suspension. In each well, 250 μL of enriched growth medium was inoculated with 25 μL from the dilution series and one well with sterile water to serve as negative controls. Aerobic plates were incubated in ambient air, and anoxic plates were incubated under anoxic conditions in air-tight containers with AnaeroGen pouches (Thermo Fisher Scientific, Walthan, MA, United States) to generate an anoxic atmosphere (Supplementary Figure S1). For each target group (aerobic Sb or Cu tolerators, fermenting Sb or Cu tolerators, Sb or Cu respirers) 8 tubes (1 per environmental sample) and 4 plates (1/2 plate per environmental sample) were prepared. All tubes and plates were incubated at 4°C for three weeks or until turbidity was observed and transferred to new tubes or plates. After three rounds of transfers, 35 tubes and 85 wells on the plates presented turbidity (from now on called enrichments) and the supplemented Sb(III), Sb(V) and Cu(II) were dissolved in the media. and 100 μL of growth medium was transferred to solid media using the spread plate technique. Plates were incubated at 4°C for three weeks or until colony growth was observed (Supplementary Table S4), and colonies were transferred onto fresh plates at least three times using the streak plate technique in order to isolate them. Isolates were tested in 96-well plates at different temperatures and concentrations to study their growth. The media used for the plates varied according to the isolates and was prepared using the same conditions as mentioned before. Growth was tested at five temperatures (0°C, 5°C, 10–15°C, 20°C, 30°C) in with final concentrations of 100 μM of Sb(V) or 1 mM of Cu(II) in the medium. Impact of concentration was tested with three concentrations of Sb and Cu (“low” = 100 μM, “medium” = 1 mM and “high” = 10 mM) at room temperature. In each case, isolates and a negative control (without microbial inoculation) were inoculated in triplicates. Microbial growth was measured regularly on the spectrophotometer (absorbance 600 nm).

### Sequencing and sequencing analyses

To study microbial communities present in the environment and enrichment, nucleic acids from the samples were co-extracted using an optimized protocol that mitigates inhibitor effects (Lim et al., 2016). For environmental samples, extraction occurred within one month of sampling, targeting for DNA and RNA to evaluate total and active microbial communities respectively, as done in previous ecological studies (Baldrian et al., 2012; Zhang et al., 2014, 2021). Conversely, extraction of enrichment samples was conducted immediately prior to their transfer to solid media, focusing solely on DNA. Each of the co-extracted samples was divided into two eppendorfs that were stored at −20°C and − 80°C to later on purify the DNA or RNA. To remove RNA from the DNA samples, a RNase treatment was performed using a diluted RNase (100 μg/mL) (Thermo Scientific^™^) and to eliminate genomic DNA from the RNA samples a DNase treatment was done using DNase Max Kit (Thermo Scientific^™^). Complementary DNA (cDNA) [qScript^™^ cDNA synthesis Kit (Quantabio)] was generated in the case of RNA samples. The V3-V4 region of the 16S rRNA gene was amplified from DNA and cDNA using universal primers 341F (CCTACGGGNGGCWGCAG) and 805R (GACTACHVGGGTATCTAATCC) (Herlemann et al., 2011) and sequenced on an Illumina MiSeq platform with 300 bp paired-end reads at Macrogen Europe. Regarding the isolates, DNA was extracted from the isolates using NucleoSpin DNA Microbial 740235.50 from MACHEREY-NAGEL^©^. After the extraction, DNA was amplified via 16S rRNA gene PCR using universal primers 27F and 1492R 27F (AGAGTTTGATCCTGGCTCAG) and 1492R (TACGGYTACCTTGTTACGACTT) (Lane, 1991) and sequenced via sanger sequencing at a commercial laboratory (Macrogen Europe). Raw sequence data of the environmental and enrichment samples were deposited into the NCBI Sequence Read Archive (SRA) under BioProject accession PRJNA1079661 and sequencing data of the isolates into NCBI GenBank under project accession PP448086-PP448136.

For the environmental and enrichment samples, diversity profiling analysis were performed with QIIME 2 2022.2 (Bolyen et al., 2019). The demultiplexed raw reads were primer trimmed, quality filtered, denoised and merged with DADA2 [Divisive Amplicon Denoising Algorithm 2 (Callahan et al., 2016)] with truncation length of 270 and 220 and trimmed length of 16 and 21 for the forward and reverse read sequence, respectively. Taxonomy was assigned to the ASVs (Amplicon Sequence Variants) using the classifier SILVA 138 (Quast et al., 2012). Phyla names were given according to the most updated taxonomy classification rule (Oren and Garrity, 2021). For each sample, phyla and genera above and below 5% abundance were considered “high” and “low” relative abundance, respectively. Rarefaction depth was settled at 21177 reads to ensure all samples were included in the analysis (reads per sample ranged from 21177 to 58267). Artifacts from QIIME2 were exported to R (version 4.2.3 2023-03-15) and a phyloseq object was created via the R package QIIME2R and phyloseq (v0.99.20 and v1.42 respectively). Enrichment and environmental sequencing data was analyzed with R packages ape (v5.7–1), ddplyr (v1.1.1), lme4 (v1.1–32), tidyr (v1.3.0), vegan (v2.6–4) and tidyverse (v2.0.0). R package microbiome (v1.21.1) and picante (v1.8.2) were used to study Faith Phylogenetic Diversity, Observed ASVs (species richness), Rarity (log_modulo_skewness) (species richness of the less abundant species) and Shannon Diversity. Forward and reverse sequences of the isolate samples were aligned with MEGA X software version 10.0.5. Neighbor-sequence alignment of the isolates was done in SILVA ACT (FastTree program, 0.95 min. Identity with query sequence) (Quast et al., 2012). The alignment was exported and a phylogenetic tree was constructed with maximum likelihood and using the Maximum Likelihood method and Tamura-Nei model (Tamura and Nei, 1993) and Neighbor-Join and BioNJ algorithms in MEGA X (Kumar et al., 2018). Most abundant ASVs of the enrichments as well as isolate sequences were also classified according to next-cultured relative in BLAST search using the NCBI database (Altschul et al., 1990). In addition, Isolate sequences were aligned with their respective enrichment and environmental sample to search for possible matches (identity >99%). For the heatmap, the tree was exported from SILVA ACT and modified (branch.length = ‘none’) in R [ggtree (v.3.6.2)]. Isolate sequences were also aligned with their respective enrichment and environmental sample to search for possible matches (identity >99%). Statistical analyses of the environmental parameters of the samples were done in R. T-test was used to find significantly different values from the mean of each parameter and ANOVA test was used to find significantly differences in the values obtained from each site. Significant *p*-values were considered from * ≤ 0.1, ** ≤ 0.01, and *** ≤ 0.001. All figures were generated in R [packages ggplot 2 (v3.4.2), ggVennDiagram (1.2.2)] and edited with Adobe Illustrator 2022.

## Results

### Sampling and physicochemical analyses of environmental samples

A total of 18 environmental samples were obtained in the summer (8) and winter (10) (Table 1). Differences in the environmental parameters of the samples were statistically tested (Supplementary Table S1). When comparing each sampling site, no significant differences were found in pH and As concentrations, but significant differences were found in Sb, Cu, Fe and S concentrations. The pH was near-neutral in site A and C, while more variation was found in each of the cores of site B. Antimony was nearly only found in site C with concentrations 75 times higher than the mean of the other sites, copper concentrations were significantly higher in site B, with an average three times higher than the mean of the other sites and As concentrations were relatively similar in all sites.

**Table 1:** Physico-chemical characteristics of environmental samples used in the study.

Site	Season	Depth (cm)	Core	pH	Concentration (mg·kg_dw_^−1^)			Concentration (g·kg_dw_^−1^)	
					Sb	Cu	As	Fe	S
A	Summer	15	1	7.4	2.9	360	1200***	58**	18**
	Summer	32	1	7.3	0.53	280*	310	68*	26**
	Summer	46	1	7.8	0.21	200**	230*	87*	37*
	Winter	< 10^a^	1	6.9	< 2	36***	63***	21***	0.79**
	Winter	15	1	6.9	< 2	270	580*	45**	7.1**
	Winter	25	1	7	< 2	200**	460	58**	16**
	Winter	35	1	7	< 2	220	700***	66*	21**
B	Summer	10	1	5.8	7.1	570**	440	190*	200*
	Summer	30	1	7.6	4.9	600**	430	220**	220**
	Summer	57	1	7.4	4.9	410	280*	120	120
	Summer	27	2	12.3***	4.0	630**	360	190*	200*
	Winter	38	1	2.5***	4.1	800***	230*	120	120
	Winter	48	1	2.3***	2.2	600**	330	370***	410***
	Winter	< 5^a^	2	12.1***	2.1	450	250*	290***	310***
	Winter	20	2	7.1	2.2	630**	250*	240**	240**
	Winter	40	2	3.8***	3.5	500*	330	240**	250***
C	Summer	10	1	7.5	240***	9.4***	190**	60*	1.3**
	Winter	10	1	7.6	240***	28***	650**	0.27***	6.5**

*p*-values are indicated as * ≤0.1, ** ≤0.01, and *** ≤0.001. "^a^" denotes the frozen layer, primarily consisting of snow.

### Microbial communities from mining environments

DNA and RNA were extracted from 18 environmental samples for 16S rRNA amplicon sequencing. A total of 5,619 ASVs were detected in all environmental samples. Most of the ASVs were detected exclusively in site C (79%), followed by site A (7.9%) and site B (7.1%). Some ASVs were detected in multiple sites, such as A and C (4.5%), A and B (0.89%) and site B and C (0.11%). In addition, 18 ASVs were detected in all sampling sites (0.32%) (Supplementary Figure S2).

**Figure 2:**
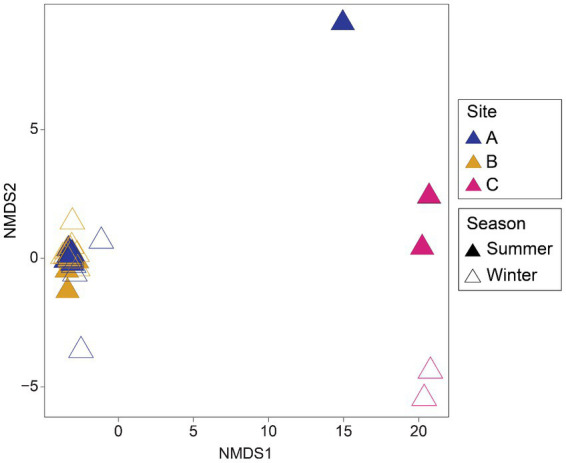
Nonmetric multidimensional scaling (NMDS) ordination of microbial assemblages from environmental samples by sites and seasons of sampling. Plot based on Bray-Curtis distances calculated from rarefied ASV tables.

Differences were found in the microbial community structure of the environmental samples when analyzed by nonmetric multidimensional scaling (NMDS) (Figure 2). Samples from site A and B form a tight cluster, whereas samples from site C exhibit a more dispersed pattern with a slight seasonal clustering effect.

**Figure 3:**
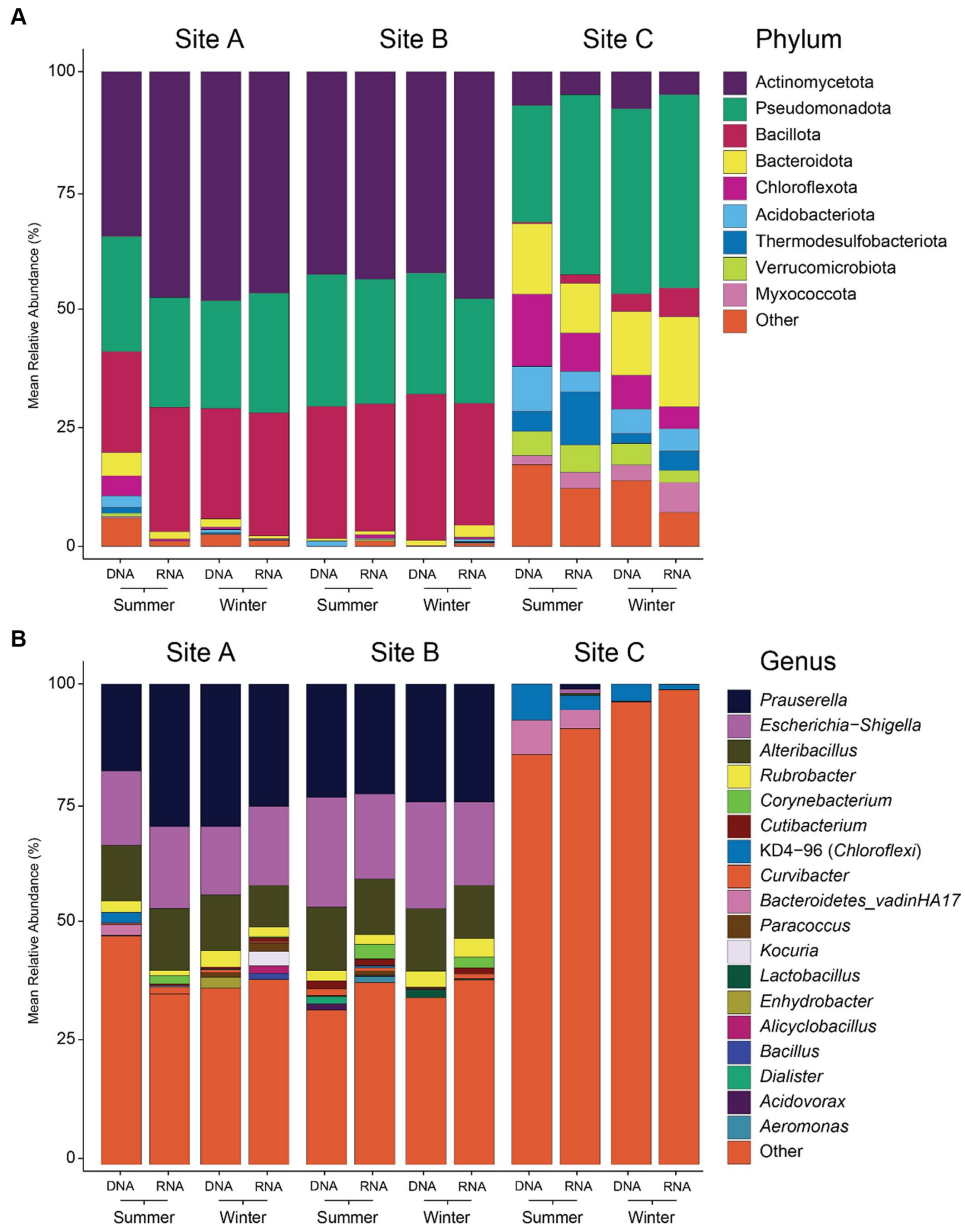
Microbial composition of environmental samples at phylum **(A)** and genus **(B)** level. Phyla and genera with a mean relative abundance of less than 5% were grouped in “Other.” Taxonomy classification based on 16S rRNA amplicon sequencing and SILVA database.

Microbial diversity was significantly higher in samples from site C than from the other two (Supplementary Tables S2, S3). Observed ASVs from site C were 28 times higher than the average of sites A and B, Shannon diversity was two times higher, rarity index 49 times higher and phylogenetic diversity 19 times higher. In contrast, no significant differences were found for microbial diversity in different seasons (summer vs. winter), depth (frozen vs. surface vs. middle vs. bottom) or nucleic acid type (DNA vs. RNA) at each site. Differences between the sites were also reflected in the taxonomic composition of the microbial communities (Figure 3 and Supplementary Figure S3). In site A and B, Actinobacteriota was the dominant phylum (44% in both sites), followed by Bacillota (24 and 28%, respectively) and Pseudomonadota (24 and 25%, respectively), while in site C Pseudomonadota was the most abundant phylum (36%), followed by Bacteroidota (14%) and Chloroflexota (9%). Genera like *Corynebacterium* and *Kocuria* were only found in seven RNA samples from sites A and B but not in site C. The representation of the low-abundant phyla was <3% in samples from sites A and B and 13% in samples from site C. When looking at genus level, differences in sites A and B were slightly more pronounced than at phylum level. Dominant genera in sites A and B were *Prauserella* (22%), *Escherichia*-*Shigella* (16 and 20% respectively) and *Alteribacillus* (11 and 12%) but most of genera were of low abundance (40 and 36%). In contrast, in site C almost all the genera (93%) were those with low relative abundances.

### Enrichment of Sb-/Cu- tolerant microbes from mining environments

Environmental samples from the summer were inoculated to tubes and 96-well plates with different enrichment media. A total of 771 ASVs were obtained from sequencing the enrichment samples, but none were found in all enrichments. Some ASVs identified in the enrichments (122, 16%) were also present in the environmental samples, while the majority of ASVs (649, 84%) were exclusively detected in the enrichments. ASVs shared between environmental and enrichment samples belonged mostly to phylum Bacillota, Pseudomonadota and Actinomycetota (Supplementary Figure S4).

**Figure 4:**
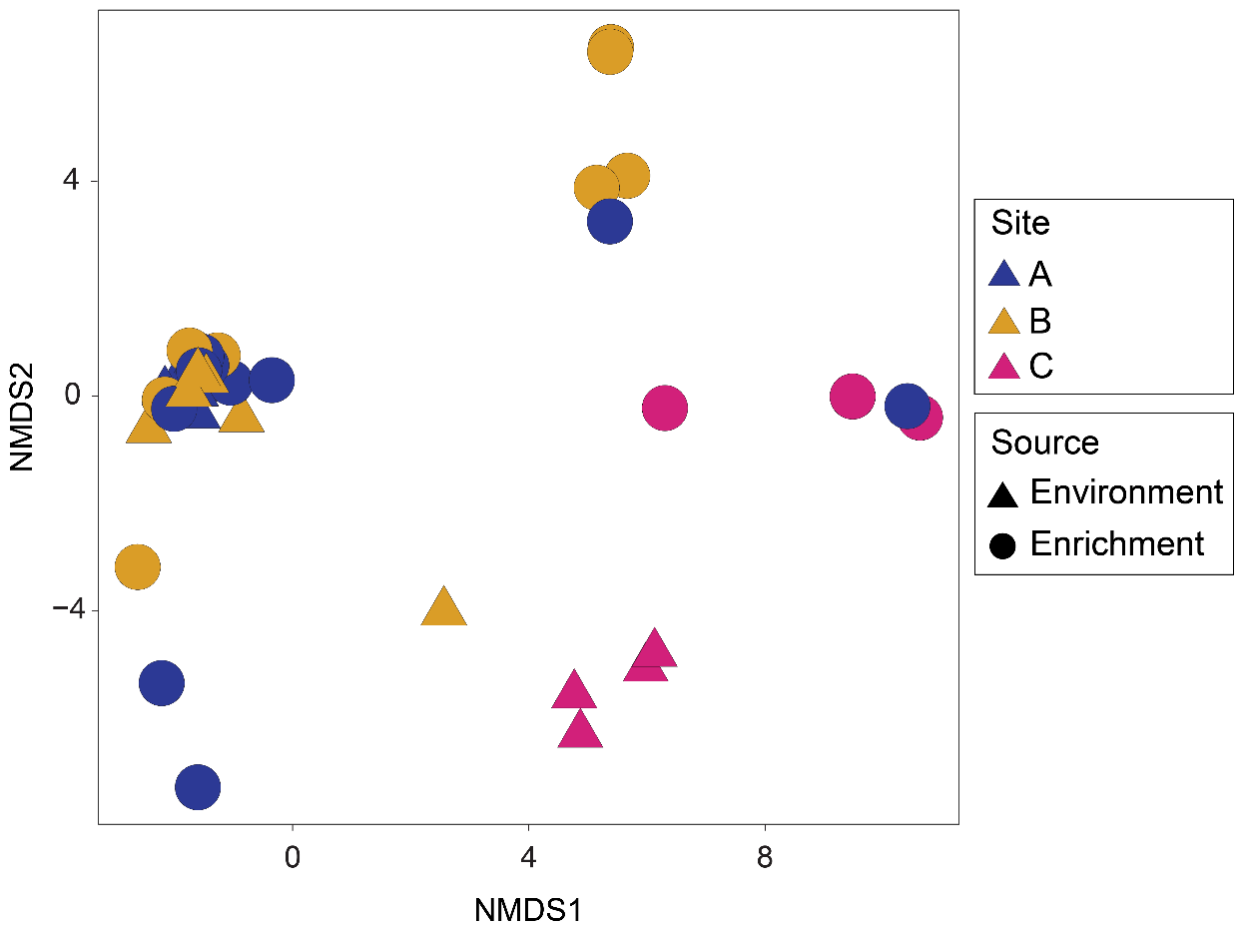
Nonmetric multidimensional scaling (NMDS) ordination of microbial communities from environmental (DNA and summer) and enrichment samples. Plot based on Bray-Curtis distances calculated from rarefied ASV tables.

Microbial community structure (Figure 4) revealed a subtle distinction between the environmental and enrichment samples from site C. In addition, there appeared to be an overlap between some environmental and enrichment samples from site A and B.

**Figure 5:**
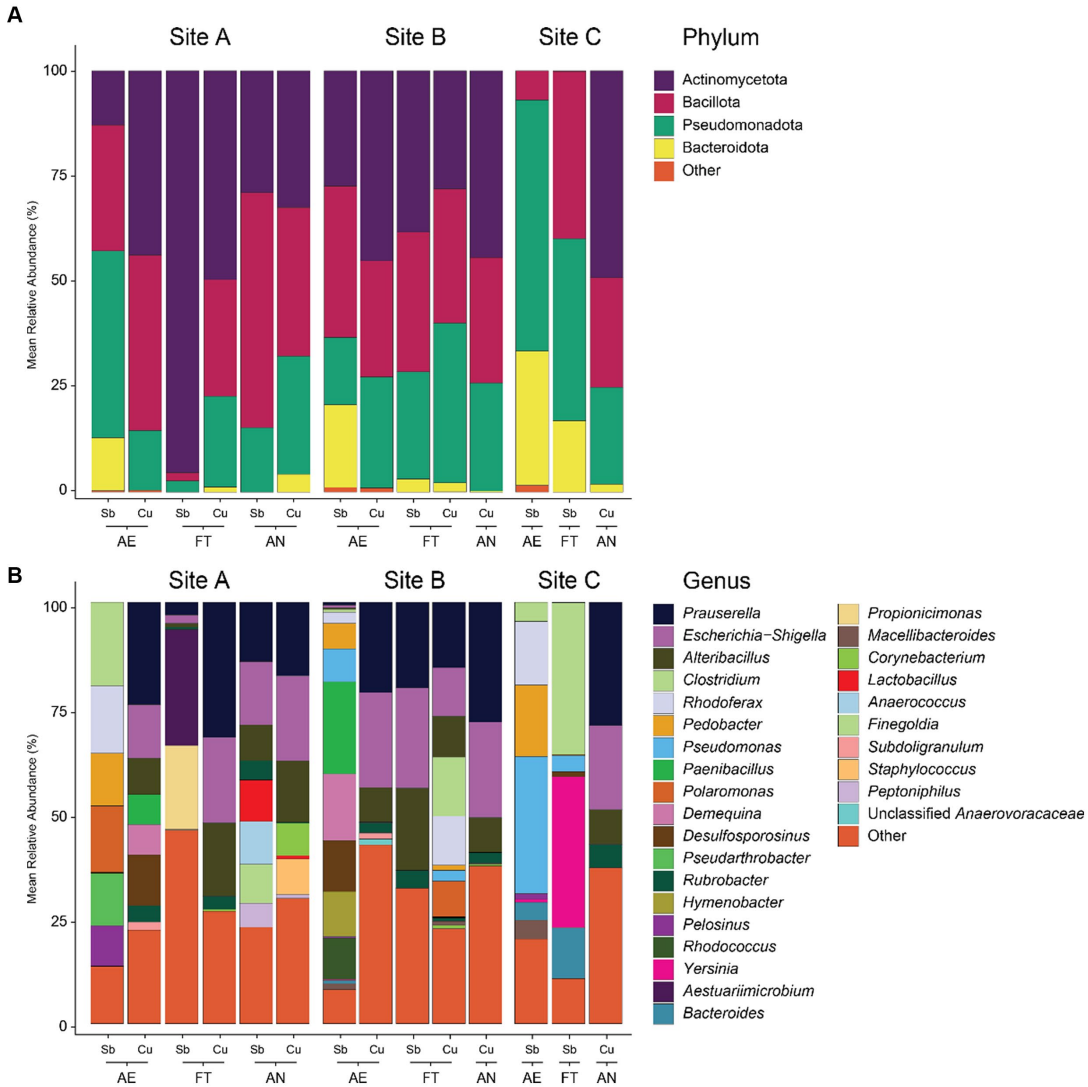
Microbial community composition of enrichment samples at phylum **(A)** and genus **(B)** level. Aerobic tolerators are described as AE, fermenting tolerators as FT and anaerobically respiring as AN. Antimonite (1 mM) was used for the cultures of aerobic antimony-tolerant microorganisms, antimonate (1 mM) for the fermenting antimony-tolerators and anaerobical antimonate respirers and copper sulfate (1 mM) for the aerobic and fermenting copper-tolerators as well as anaerobic copper- or sulfate-reducer. Phyla and genera with a mean relative abundance of less than 5% were grouped in “Other.” The genera “Clostridium_sensu_stricto_13” and “Clostridium_sensu_stricto_9” were grouped together as “*Clostridium*.” Taxonomy classification based on 16S rRNA amplicon sequencing and SILVA database. The microbial composition presented in this figure represents the results of three rounds of enrichment transfers, with each round involving 3 weeks of culturing.

Considering all the enrichment samples (except the anaerobic Sb-cultures, whose sequencing was not carried out), differences were found in the microbial composition of the enrichments according to different sites (Figure 5 and Supplementary Figure S3). In enrichments from sites A and B, Actinobacteriota was the dominant phylum (35 and 36% respectively), followed by Bacillota (32 and 31%, respectively) and Pseudomonadota (27 and 26%, respectively), while in enrichments from site C the dominant phylum was Pseudomonadota (46%), followed by Bacteroidota (21%), and Bacillota (20%). On genus level, enrichments from sites A and B were dominated by *Prauserella* (12 and 16%, respectively) and *Escherichia*-*Shigella* (8 and 16%, respectively) but most of the taxa belong to genera with relative abundances <1% (22 and 28%, respectively). In contrast, the most abundant genus in enrichments from site C was *Pseudomonas* (17%), followed by *Clostridium* (11%) and *Yersinia* (9%) and most genera had a relative abundance <1% (22%). *Corynebacterium*, which was abundant in environmental samples, was only found in small proportions in enrichments from sites A and B while *Kocuria* was not detected in any of the samples.

**Figure 6:**
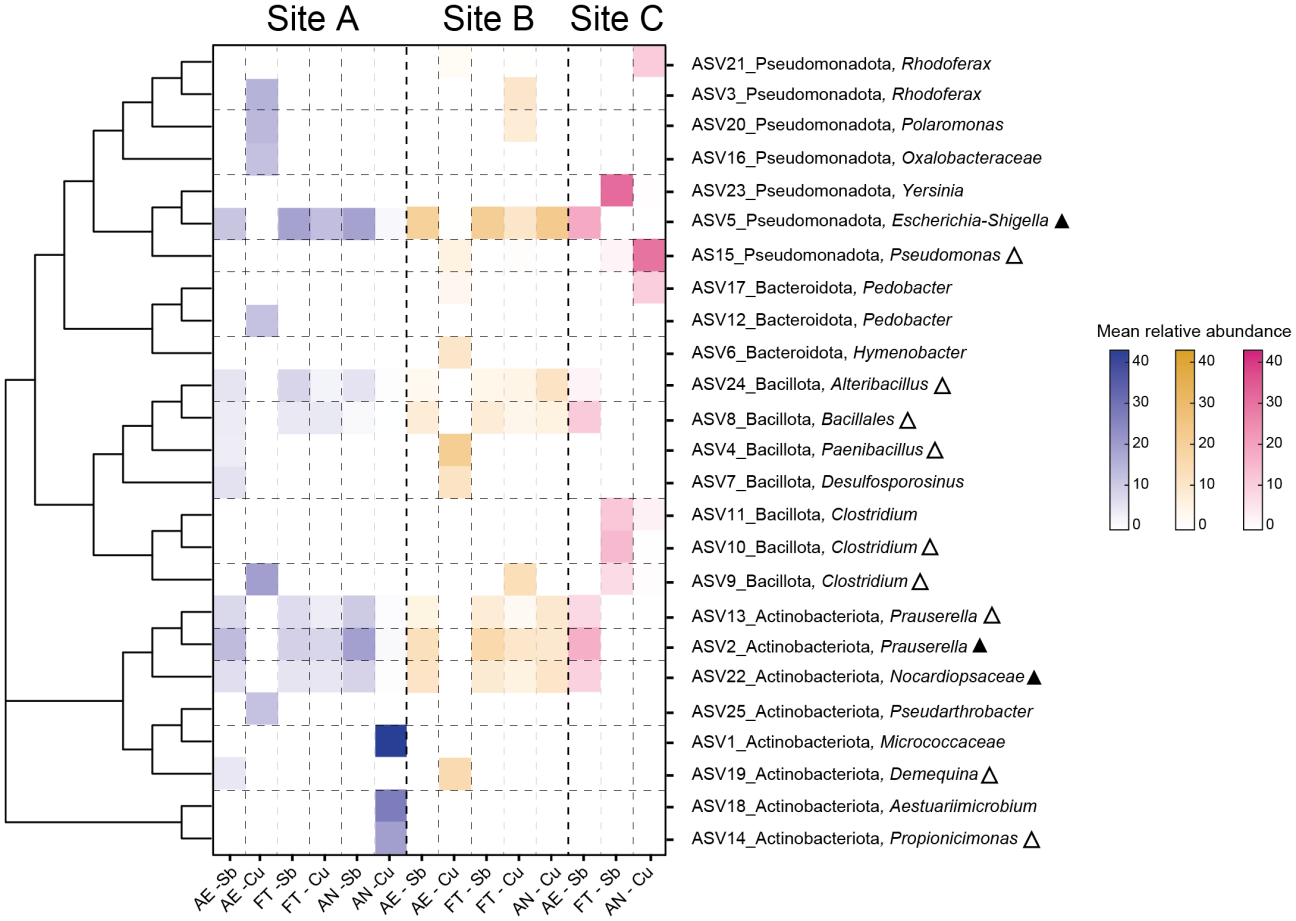
Heat map representing dominant ASVs (>10% mean relative abundance) in the enrichments. Aerobic tolerators are described as AE, fermenting tolerators as FT and anaerobically respiring as AN. Triangles next to the taxa indicate that the ASV is also present in at least one of the environmental samples (blank triangle) or that the ASV is dominant in at least one of the environmental samples (filled triangle).

In the enrichments, ASVs with >10% mean relative abundance were considered dominant taxa. Almost half of the 25 dominant ASVs (12) were detected in the environmental samples, and three of them were considered abundant in at least one of the environmental samples (Figure 6). Dominant ASVs (based on SILVA taxonomy classification) were plotted in a heatmap, and a phylogenetic tree was added to infer phylogeny. Next-cultured relative from BLAST search is found in Supplementary Table S5.

### Isolation of putative Sb and Cu-metabolizing isolates

**Figure 7:**
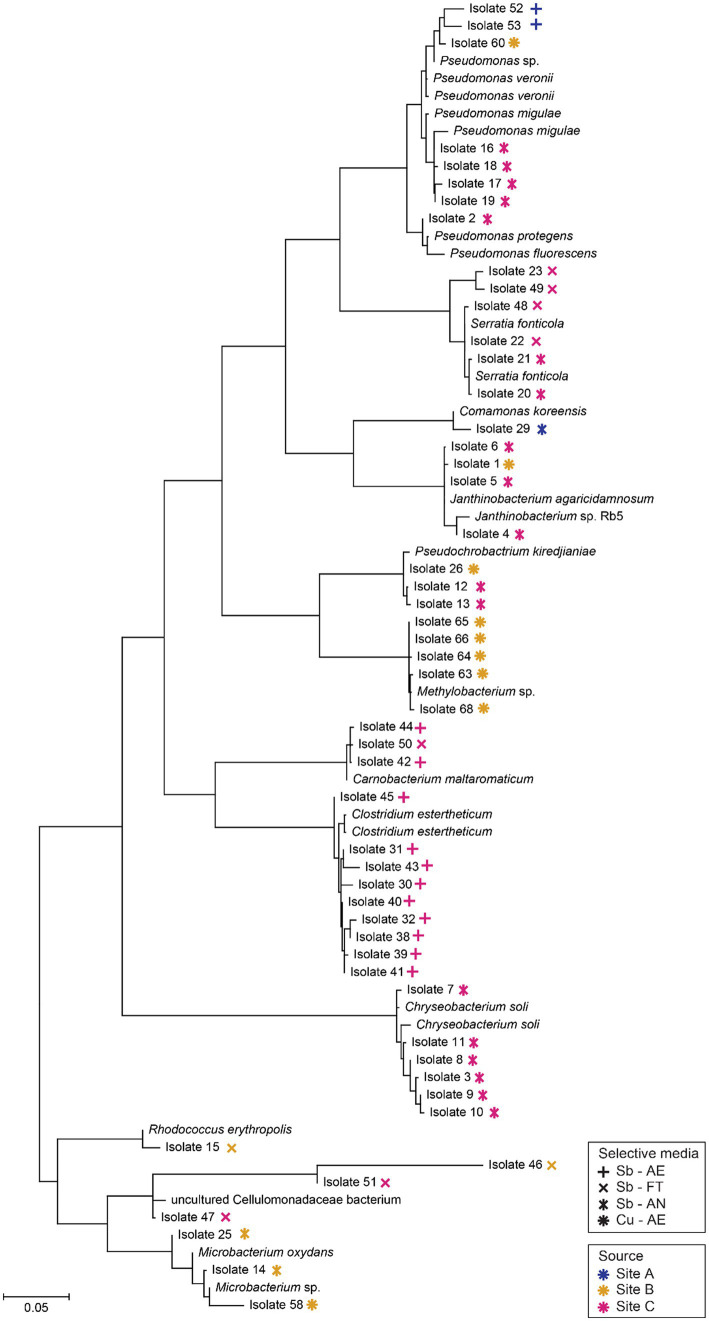
Phylogenetic tree of 16S rRNA gene sequences of putative Sb-/Cu-metabolizing bacterial isolates (according to their enriched media and site of origin) and their closest neighbors. Sequencing alignment was done in SILVA and tree was constructed in MEGA X.

A total of 65 putative Sb and Cu-metabolizing microbes were isolated from the enriched samples. Based on the media of isolation, 26 isolates were aerobic -tolerators, 9 Sb-fermenting tolerators, 18 anaerobic Sb-respirers and 12 aerobic Cu-tolerators. No fermenting Cu tolerator or anaerobic Cu- or sulfate-reducer was obtained. Sequences of 13 isolates were not obtained due to low results of amplification and/or bad sequencing quality. 52 isolates were successfully identified based on their 16S rRNA gene sequences and mapped onto a phylogenetic tree (Figure 7). Most isolates sequences (31) revealed less than 99.2% identity to cultured representatives and few of them (4) showed less than 97.5% (Supplementary Table S6). The majority of isolates were associated with phyla Pseudomonadota (26), Bacillota (13), Actinomycetota (7) and Bacteroidota (6). Many of the Bacillota isolates came from the anaerobic enrichments [fermenting Sb-tolerators (1) and anaerobic Sb-respirers (11)] and most of the Pseudomonadota isolates came from the aerobic enrichments [aerobic Sb-tolerators (14) and aerobic Cu-tolerators (6)]. Bacteroidota were only isolated in aerobic Sb-tolerator enrichments.

Regarding the alignments between the isolates and environmental samples, ten isolates from site C matched at least one of the ASVs of their respective environmental sample, but no matches were found between the isolates from sites A and B and their environmental sample. Concerning the alignments between the isolates and the enrichment samples, nine isolates matched at least one of the ASVs from their respective aerobic Sb-tolerators enrichment and six isolates from the fermenting Sb-tolerators enrichment. No isolates matched an ASV from both, their respective enrichment and environmental sample (Supplementary Table S6).

The impact of temperature and Sb or Cu concentration on growth was determined for 63 isolates. While all the isolates demonstrated at least partial growth at the lowest temperature and the majority (70%) exhibited growth at the highest temperature, neither the lowest nor the highest temperatures were optimal for any of them. Most of the aerobic isolates (76%) had an optimum temperature of 20°C, in contrast with the anaerobic isolates, most of which (95%) had an optimum temperature below 20°C. All isolates were capable of growing in the smallest Sb or Cu concentration (0.1 mM) and most of them (81%) grew as well in the highest concentration (10 mM), but the preferable Sb or Cu concentration for the majority of them (79%) was the lowest one.

## Discussion

### Biotechnology potential of putative Sb-/Cu-tolerant cold-adapted microorganisms

There is an increasing interest in studying microbial communities from mining contaminated sites because of their ability to transform metal(loid)s and their potential in industrial applications (Levett et al., 2021; Newsome and Falagán, 2021; Staicu and Stolz, 2021). One approach of Sb-bioremediation consists of Sb(V) reduction and subsequent precipitation of the formed Sb(III) (Wang et al., 2013, 2018; Zhu et al., 2018). However, only few Sb-resistant species have been isolated, and much more is known about Sb(III)-oxidizing than about of Sb(V)-respiring microorganisms (Li et al., 2019; Shi et al., 2019; Chen Y. et al., 2022). This lack of knowledge is especially the case for Sb-resistant psychrophilic and psychrotolerant microorganism, which are of particular interest for their *in-situ* applicability in bioremediation systems of Arctic mining environments. In this study, 65 putative Sb- and Cu- metabolizing microorganisms from subarctic mine sites were enriched and isolated at 4°C in three different media selecting for: aerobic tolerators, fermenting tolerators and anaerobically respiring microorganisms. Considering that previous studies have determined the values of Sb(III) and Cu(II) that inhibit microbial growth of model bacteria such as *Escherichia coli* (Yamamoto and Ishihama, 2005; An and Kim, 2009) to be >0.1 mM for Sb(V) and > 1 mM for Cu(II), it is therefore asserted that all the microorganisms isolated in this study exhibit a certain degree of resistance to Sb or Cu, as they were all capable of growing at concentrations higher than 0.1 mM and a portion of them even at 10 mM. While no tests were conducted to assess the metabolic capabilities of the candidates, few hints suggest their potential for Sb/Cu-metabolizing abilities. For instance, the color of certain colonies changed after being inoculated to plate or a halo surrounding colonial growth appeared after inoculation (Supplementary Figure S1). Also, although the final enrichments for putative Sb-fermenting tolerators and putative anaerobically Sb-respiring microorganisms were supplemented with Sb(V), no growth was observed when supplementing them with Sb(III) (Supplementary Table S4). In addition, the metal(loid)s supplemented in the media, including the Sb(V) which took longer than the rest and whose particles were initially visible, were dissolved in all the liquid culture after weeks of inoculation, indicating there is a biochemical change occurring in the cultures. Therefore, while not the primary focus of this paper, several indications throughout the enrichment and isolation processes suggest the potential of the isolated microbes to utilize Sb(V) as an electron acceptor or Cu(II) as part of their metabolism. Considering the challenging environmental conditions of Arctic mines in terms of temperature and metal(loid) concentrations, microorganisms isolated in this study emerge as promising candidates for bioremediation applications within *in-situ* systems of (sub-)Arctic mines. Further tests with the isolates are needed to confirm their metabolizing capabilities and feasibility in the system but findings from this study represent the first step in the process to establish a metal(loid) immobilization system under low temperatures.

### New metal(loid)-tolerance insights into known environmental microorganisms

At taxonomic level, most of the putative anaerobic Sb(V)-respiring isolates were related to *Clostridium estertheticum,* an anaerobic bacterium which was firstly isolated from vacuum-packed beef (Collins et al., 1992) and was classified as a true psychrophile when isolated from an Antarctic microbial mat (Spring et al., 2003). When the genome of *C. estertheticum* was studied (Yu et al., 2016), metal-resistance genes were found. It is particularly interesting the identification of *arsBDR* and *pstB,* genes which confer resistance to As and Sb (Carlin et al., 1995; Li et al., 2016), two metalloids that are often associated in the nature. However, from our knowledge, no *C. estertheticum* has been isolated from Sb-respiring cultures. Apart from the genus *Clostridium*, the other putative anaerobic Sb-respiring isolates were related to the genus *Pseudomonas*. *Pseudomonas* spp. have been reported for their ability to reduce a wide variety of metals (Blake et al., 1993), potentially including the reduction of Sb(V) (Lai et al., 2016; Sun et al., 2021). In particular, one of the isolates was related to *Pseudomonas veronii,* a metal tolerant species of this genus, which has been studied for bioremediation (Malla et al., 2018) and whose genome has been found to have the heavy metal resistance gene arsBDR (Morales et al., 2016). Furthermore, As-tolerant *Pseudomonas* strains have been previously isolated from same location as site C (Ziegelhöfer and Kujala, 2021). However, unlike the strains presented in this study, the previously reported *Pseudomonas* showed an optimum growth at 20–28°C, implying this might be a different isolated strain as the optimum temperatures for the *Pseudomonas* presented here was 10°C. Finally, two isolates were related to *Carnobacterium maltaromaticum* with a high identity percentage. This species has been described as a facultatively anaerobic fermenting bacteria (Rainey et al., 2009) and its tolerance to other metals such as Cd have been assessed (Butrimienė et al., 2022), but not its interactions with Sb. Overall, these results open a gap for further bioremediation studies as the isolates, particularly those from the anaerobic Sb-respiring tolerators, might have the potential to be used in bioremediation by using Sb(V) as an electron acceptor.

Given the limited number of species that have been described for Cu-reducing capabilities (Kimber et al., 2023), the anaerobic Cu-enrichment cultures prove to be especially relevant. Even though growth was observed in different Cu-enrichment cultures (Supplementary Table S4), only few Cu microorganisms were isolated on plates, which might be explained by “great plate count anomaly” in which the number of microorganisms is reduced when transferring them to solid media (Staley and Konopka, 1985). Overall, most of the Cu-isolates were psychrotolerant which is expected as usually, more psychrotolerant than psychrophilic species are isolated from permanently cold environments (Männistö and Puhakka, 2002). Since temperature can affect the behavior of microbial communities in the environment, becoming the limiting factor in bioleaching applications (Dopson et al., 2007; Xiao et al., 2017), efforts are being made in order to characterize bioleaching bacteria which can carry out its activity at low temperature and being therefore a good candidate for mining areas with low mean temperatures (Muñoz-Villagrán et al., 2022). While some of the isolated strains were able to grow at temperatures near 0°C, their Cu-resistance was low (1 mM) in comparison with strains isolated for biomining in previous studies (>100 mM) (Orell et al., 2010; Barahona et al., 2020). It is therefore suggested that the putative Cu-metabolizing microorganisms isolated might not be good candidates for bioleaching applications. Nevertheless, because of the well-known advantages of using microbial consortia for bioleaching (Brune and Bayer, 2012; Liu et al., 2020; Bobadilla-Fazzini and Poblete-Castro, 2021), microorganisms from this study could still have potential applications, particularly due to their cold-tolerance or in other systems with lower Cu-concentrations.

### Majority of environmental microorganisms are missed by selectively enrichment approach

In the present study, microorganisms isolated differed from the diversity found in the enrichment samples and environment. Differences were found in the microbial community structures of the environmental and enrichment samples (Figure 4). The most evident observation of this dissimilarity came with the total number of ASVs from the environment, which was seven times larger than the total number ASVs from the enrichments (Supplementary Figure S2). Certain phyla like Bacillota might be favored during the enrichment stage as, e.g., its abundance highly increased in site C. As expected, the abundances of Chloroflexota and Acidobacteriota, phyla which have been reported for their slow growth and difficulties for isolation (Campanharo et al., 2016; Salam et al., 2021; Sansupa et al., 2021), were reduced in the enrichments. Genera like *Pseudomonas* or *Clostridium*, which were not abundant in the environment of site C, dominated some of the enrichment samples from this site. These results were expected *Pseudomonas* is a fast-growing, metabolically versatile genus (Jones et al., 2021). In fact, *Pseudomonas* has been reported in previous studies (Ponce-Soto et al., 2015) for increasing its abundance during enrichment experiments, including in experiments from cold-metal contaminated sites (Ziegelhöfer and Kujala, 2021). Some genera like *Corynebacterium* or *Kocuria,* which were relatively high in the environmental samples from sites A and B, had reduced relative abundances in the enrichments. Both genera include several species which have been described for their slow growth, especially under cold conditions (Bernard and Funke, 2015; Youn and Seo, 2022). In both the enrichments and the environment, few taxa resulted in unclassified at genus level, including some of the most abundant ASVs of the enrichments (Figure 5), indicating that previously uncultured taxa have been selectively enriched. Our findings seem to confirm the main goal of the enrichment experiment, to selectively enrich microorganisms of interest (regardless of their abundance in the environment) by creating conditions that are favorable for their growth while not supporting growth of non-target groups was accomplished.

### Microbial communities from sub(-arctic) mine sites

Mine sites, especially those in extreme environments like arctic regions, pose challenges due to their environmental conditions, which significantly influence the growth of microorganisms. Metal(loid)s and temperature are an example of selective pressure in the environment (Ledin, 1996; Bruins et al., 2000; Hao et al., 2021; Wang et al., 2021; Rijkers et al., 2022). Therefore, the environmental low temperatures and metal(loid) concentration which characterizes the sites for this study are particularly challenging for microbial communities. In the present study, sites A and B were mine tailings and site C was a peatland treating mining-affected waters. Peatlands are carbon-rich wetland ecosystems where peat, an organic-rich soil is formed (Lourenco et al., 2023). It is well known that peatlands are the habitat of a wide diversity of microorganisms with different metabolic activities, including those affected by metal(loid)s (Preston et al., 2012; Andersen et al., 2013; Kujala et al., 2020). Chodak and Niklińska (2010) found that in post-mining environments, the texture of the soil was the main factor shaping microbial communities, where loamy sands supported more microbial activity than sandy soils. Microbial composition exhibited significant variations primarily across different sites. Specifically, microbial communities from site C (water treatment peatland) exhibited markedly higher diversity than those from sites A and B (tailings). Differences between sites A and B, as opposed to site C, were also distinguished when observing the taxonomy. For example, sites A and B exhibited a dominance of three distinct phyla: Actinomycetota, Pseudomonadota, and Bacillota. In contrast, site C showed a much more even distribution of phyla in the samples, including the high representation of Chloroflexota and Bacteroidota, which is consistent with results from earlier studies on frozen peatlands (Liu et al., 2022). At the genus level, differences between sites A and B were slightly more pronounced yet notably similar when compared to the genera observed in site C. In site A and B the dominant genera were *Prauserella*, *Escherichia*-*Shigella* and *Alteribacillus*. In contrast, nearly all detected taxa in site C were represented by low-relative abundance genera, which aligns with the high values obtained in the rarity index, the indicator for the species richness of less abundant species, in site C (Supplementary Table S3). Contrary to expectations based on previous studies (Baldrian et al., 2012; Zhang et al., 2014), total (DNA) and active (RNA) microbial communities showed no variations across all samples or when grouped by sites (Supplementary Table S2 and Figure 3). In addition, difference between DNA- and RNA-based community composition appeared to be insignificant, as evidenced by the results of the CCA (Supplementary Figure S5). Considering RNA as a better approach to describe changes in active microbial communities due to, for instance, its lower stability in the environment compared to DNA (Jia et al., 2020), our results suggest certain degree of consistency for both, active and total microbial communities.

Toxic metal(loid)s were detected in the samples; in particular, site C and site B, had significantly higher values of Sb and Cu, respectively, (Table 1). When plotting the chemical elements on the CCA (Supplementary Figure S5), Sb appeared to be an important factor shaping microbial communities. However, high concentrations of Sb were only found in site C, the site from a different mining environment and soil texture. Consequently, we do believe changes in the microbial communities were not solely due to the Sb but to the texture of the soil. However, the higher diversity and Sb concentrations of site C might have played a role as most of the Sb-tolerant isolates (79.3%) originated from site C (Supplementary Table S6). In addition, higher levels of Cu were only found in site B, the one from where all of the Cu-tolerant microorganisms were isolated. However, Cu concentrations in the present study were not as high as other places from where Cu-reducing microorganisms have been isolated (Kimber et al., 2023). These results suggest that metal(loid) concentrations present in the environment might play a significant role in the isolation of tolerant microorganisms. Also, to observe the effect of the temperature on microbial communities, samples were collected from the same sites during summer and winter to observe changes in microbial community in two opposite arctic seasons. However, no significant differences in the microbial community composition were found between the summer and winter samples (Figures 2, 3). Our results support the notion that some microorganisms can be persistent and active under different temperature conditions. Gonzalez and Aranda (2023) described that in environments under changing conditions, microorganisms develop different growth states. This strategy is the reason of why certain thermophiles have been found in cold soils (Marchant et al., 2008). Thus, even if our results do not show changes in the microbial taxa detected in the summer and the winter, further studies on their growing rates could better explain the persistence of the microorganisms throughout the year.

## Conclusion and limitations of the study

This study provides valuable insights into the biotechnological potential of putative Sb and Cu-tolerant cold-adapted microorganisms isolated from subarctic mine sites. The presented putative anaerobic Sb(V)-respiring microorganisms represents, to our knowledge, the first potentially Sb-metabolizing cold-tolerant microorganisms isolated. With the increasing development of mining operations in Northern regions like Finland, recognized as one of the leading countries globally in mineral exploration (Tuusjärvi et al., 2014; Mejía and Aliakbari, 2023), the microorganisms isolated in this study, emerge as promising candidates for bioremediation applications within *in-situ* systems of (sub-)Arctic mines as the isolation of metal(loid)- and cold-tolerant microorganisms are the first step in the process to establish a metal(loid) immobilization system under low temperatures. While the putative Cu-metabolizing isolates, may not be optimal candidates for conventional bioleaching applications due to their lower Cu-resistance compared, they still exhibit Cu-tolerance and cold adaptation. A different approach during the Cu-enrichment, e.g., lowering the pH of the medium or sampling in sites where Cu concentrations were higher, could have promoted the growth strains with a higher Cu tolerance. Furthermore, changes in the microbial communities from the environment to the isolates reflect the successful implementation of an enrichment protocol and suggest that high abundances in the environment do not correspond to chances of isolation. However, considering the limitations of amplicon-sequencing (Alteio et al., 2021), it is important to remark that even if some ASVs were not detected in the environment, it does not necessarily mean that the ASV was not present. Overall, the presented findings contribute to the understanding of metal(loid)-tolerant microbial communities in subarctic mine sites and open avenues for future biotechnology applications of cold-adapted metal(loid)-tolerant microorganisms.

## Data availability statement

The datasets presented in this study can be found in online repositories. The names of the repository/repositories and accession number(s) can be found at: NCBI – PRJNA1079661, https://www.ncbi.nlm.nih.gov/bioproject/?term=PRJNA1079661.

## Author contributions

FP-F: Conceptualization, Data curation, Formal analysis, Investigation, Methodology, Software, Visualization, Writing – original draft, Writing – review & editing. SL: Conceptualization, Methodology, Resources, Software, Supervision, Validation, Writing – original draft, Writing – review & editing. KK: Conceptualization, Funding acquisition, Investigation, Methodology, Project administration, Resources, Supervision, Validation, Writing – original draft, Writing – review & editing.

## Supplementary material

SUPPLEMENTARY TABLE S1 One-sample *t*-test results for environmental parameters across different samples. 95% Confidence Interval represented as CI and Degrees of Freedom as Df.

SUPPLEMENTARY TABLE S2 ANOVA results for diversity metrics of environmental samples grouped by nucleic acid type (DNA vs RNA), site (A vs B vs C), season (summer vs winter) and depth (frozen vs surface vs middle vs bottom). Metrics were measured including all study sites, including only site A, including only site B and including only site C. Top of Form.

SUPPLEMENTARY TABLE S3 Diversity measures of the microbial communities in environmental samples.

SUPPLEMENTARY TABLE S4 Growth (turbidity) of enrichment tubes after first (1), second (2), and third (3) inoculations. Turbidity levels are indicated as *** for highly turbid, ** for medium turbid, and - for no turbidity. Sb-fermenting tolerators and anaerobically respiring tolerators Sb-enrichments went through a second round of enrichment from the first inoculation (marked as 2^a^ and 3^a^).

SUPPLEMENTARY TABLE S5 Next-related sequences of the most abundant ASVs detected in the enrichment samples. Relative abundances in the enrichments are separated by their site of origin (A, B or C) and the type of enrichment, where Aerobic tolerators are described as AE, fermenting tolerators as FT and anaerobically respiring as AN.

SUPPLEMENTARY TABLE S6 Identification of isolates using different enrichment and isolation media. Identification was made using BLAST based on the NCBI database. Growth at different temperatures and concentrations is given in categories related to their growth curves where (-) no growth observed (<5% of maximum), (+) low growth (<25% of maximum), (++) substantial growth (<75% of maximum), (+++) high growth (<85% of maximum), and (++++) maximum growth (>85% of maximum). Relative abundance indicates the abundance of the abundance of one or more than one ASV from the respective environmental and enrichment sample which matched the isolate sequence (>99% identity). ᵃ=there was more than one match but on the table is only included the one with the highest relative abundance.

SUPPLEMENTARY FIGURE S1 Pictures of enrichments and isolate cultures. One aerobic Sb-enrichment sample from the third inoculation **(A)**, isolates in anaerobic jars **(B)**, colonies of isolate 68 after a month of inoculation **(C)** colony of isolate 23 and its halo marked with an arrow **(D)**.

SUPPLEMENTARY FIGURE S2 Venn Diagram of ASVs detected in all the stages of the study, where big circles represent ASVs detected in the environmental samples from each site, medium circles the enrichment samples and small circles the isolated colonies. Blue circles are ASVs from site A, yellow circles B and pink circles C. Inside of each circle there is the total number of ASVs detected, and the overlap region of two or the three circles indicates the shared detected ASVs.

SUPPLEMENTARY FIGURE S3 Microbial composition of environmental, enrichments and control samples at genus level. Genera with a mean relative abundance of less than 10% were grouped in “Other”. Taxonomy classification based on 16S rRNA amplicon sequencing using SILVA database.

SUPPLEMENTARY FIGURE S4 Heat map representing ASVs (relative abundance >0.05) shared between environmental (Env) and enrichment (Enr) samples and its phylogenetic relationships. Site A (blue) is plotted on the first and second group of columns, B (yellow) in third and fourth and C (pink) in fifth and sixth. A cladogram of the ASVs and the results of the SILVA alignment was added next to the heatmap to infer phylogeny relationships.

SUPPLEMENTARY FIGURE S5 Canonical Correspondence Analysis (CCA) of environmental samples from different sites and seasons. Texture (tailings vs peat), genetic (DNA vs RNA) and season (summer vs winter) were processed as binary values in the metadata.
