# Supplementary figures and images for: Assessment of microbial communities from cold mine environments and subsequent enrichment, isolation and characterization of putative antimony- or copper-metabolizing microorganisms
Source: Front Microbiol. 2024 May 24;15:1386120. doi: 10.3389/fmicb.2024.1386120 (PMC11160943; doi:10.3389/fmicb.2024.1386120)

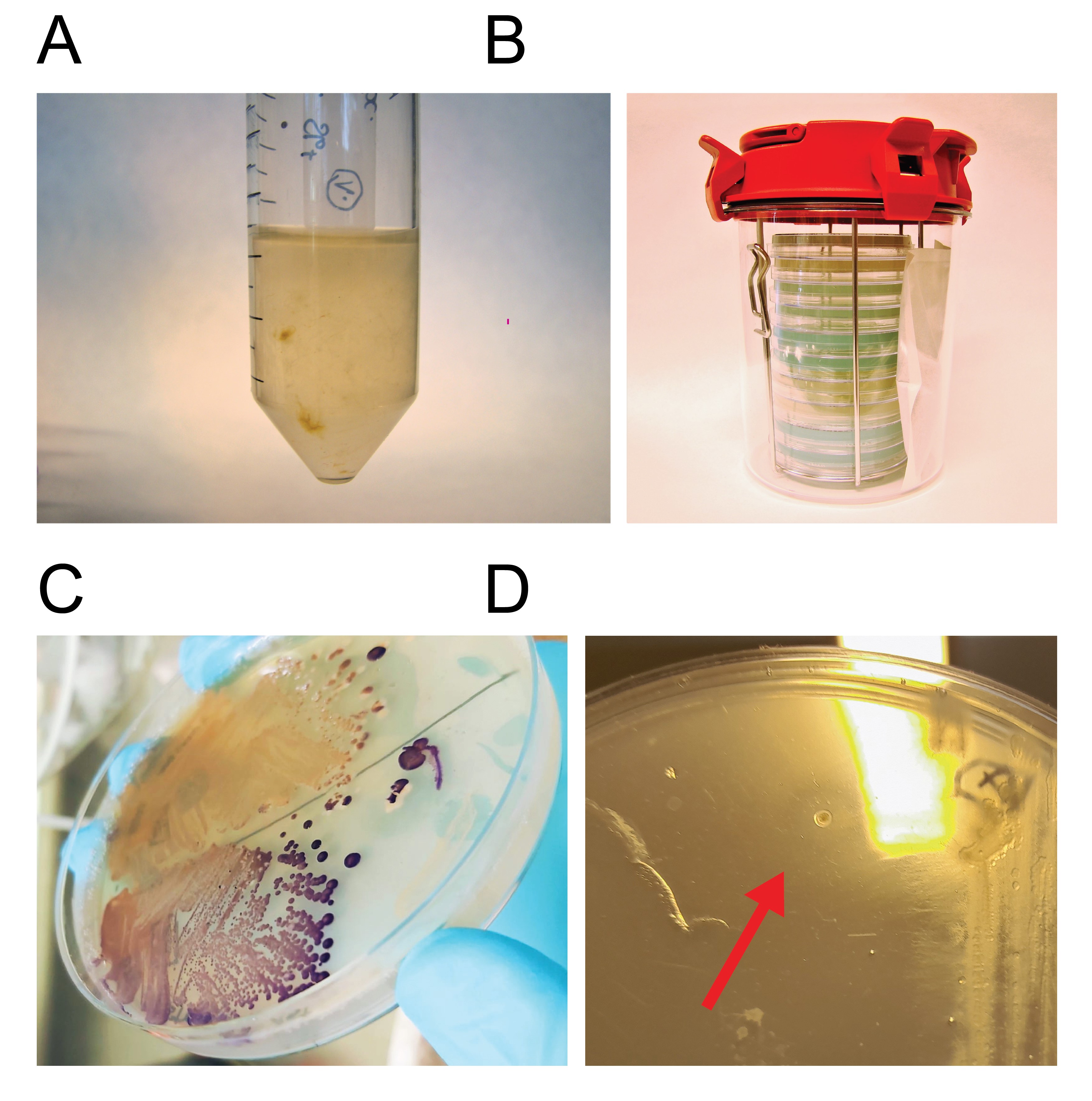

Supplement: SUPPLEMENTARY FIGURE S1 — Pictures of enrichments and isolate cultures. One aerobic Sb-enrichment sample from the third inoculation (A), isolates in anaerobic jars (B), colonies of isolate 68 after a month of inoculation (C) colony of isolate 23 and its halo marked with an arrow (D). [file Data_Sheet_2.zip › Supplementary_Figure_1.jpg]

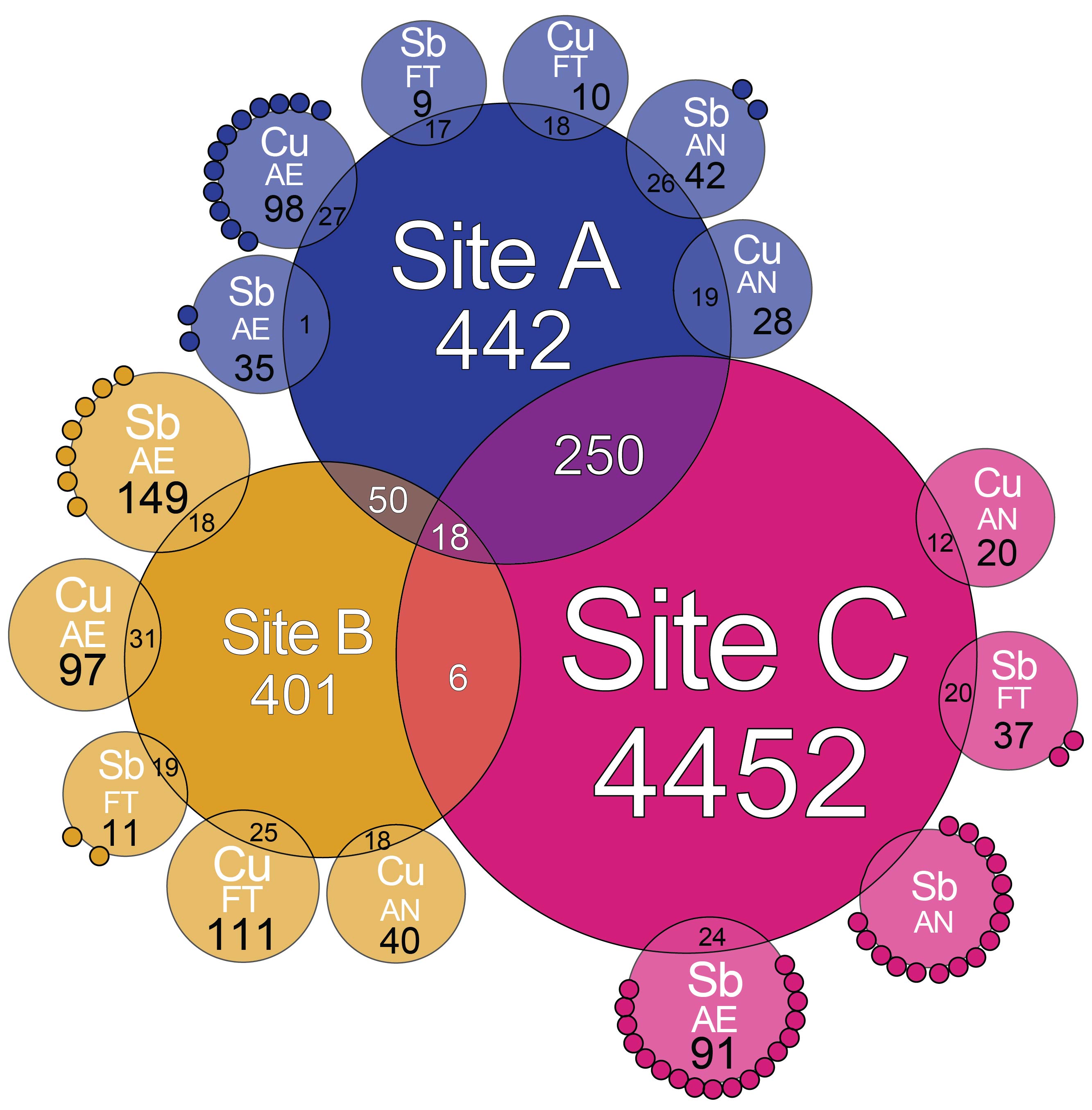

Supplement: SUPPLEMENTARY FIGURE S1 — Pictures of enrichments and isolate cultures. One aerobic Sb-enrichment sample from the third inoculation (A), isolates in anaerobic jars (B), colonies of isolate 68 after a month of inoculation (C) colony of isolate 23 and its halo marked with an arrow (D). [file Data_Sheet_2.zip › Supplementary_Figure_2.jpg]

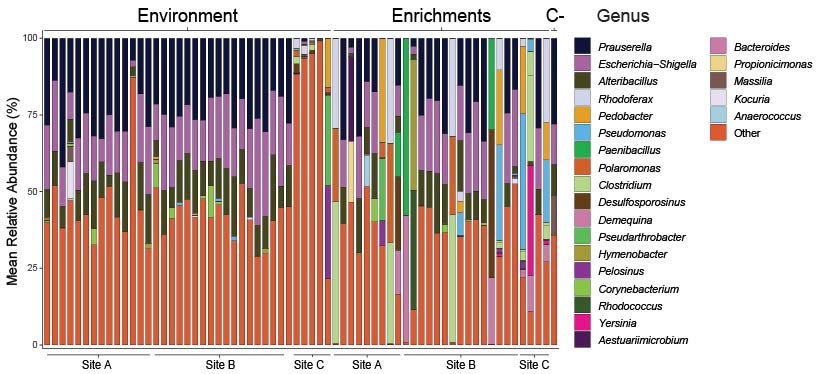

Supplement: SUPPLEMENTARY FIGURE S1 — Pictures of enrichments and isolate cultures. One aerobic Sb-enrichment sample from the third inoculation (A), isolates in anaerobic jars (B), colonies of isolate 68 after a month of inoculation (C) colony of isolate 23 and its halo marked with an arrow (D). [file Data_Sheet_2.zip › Supplementary_Figure_4.jpg]

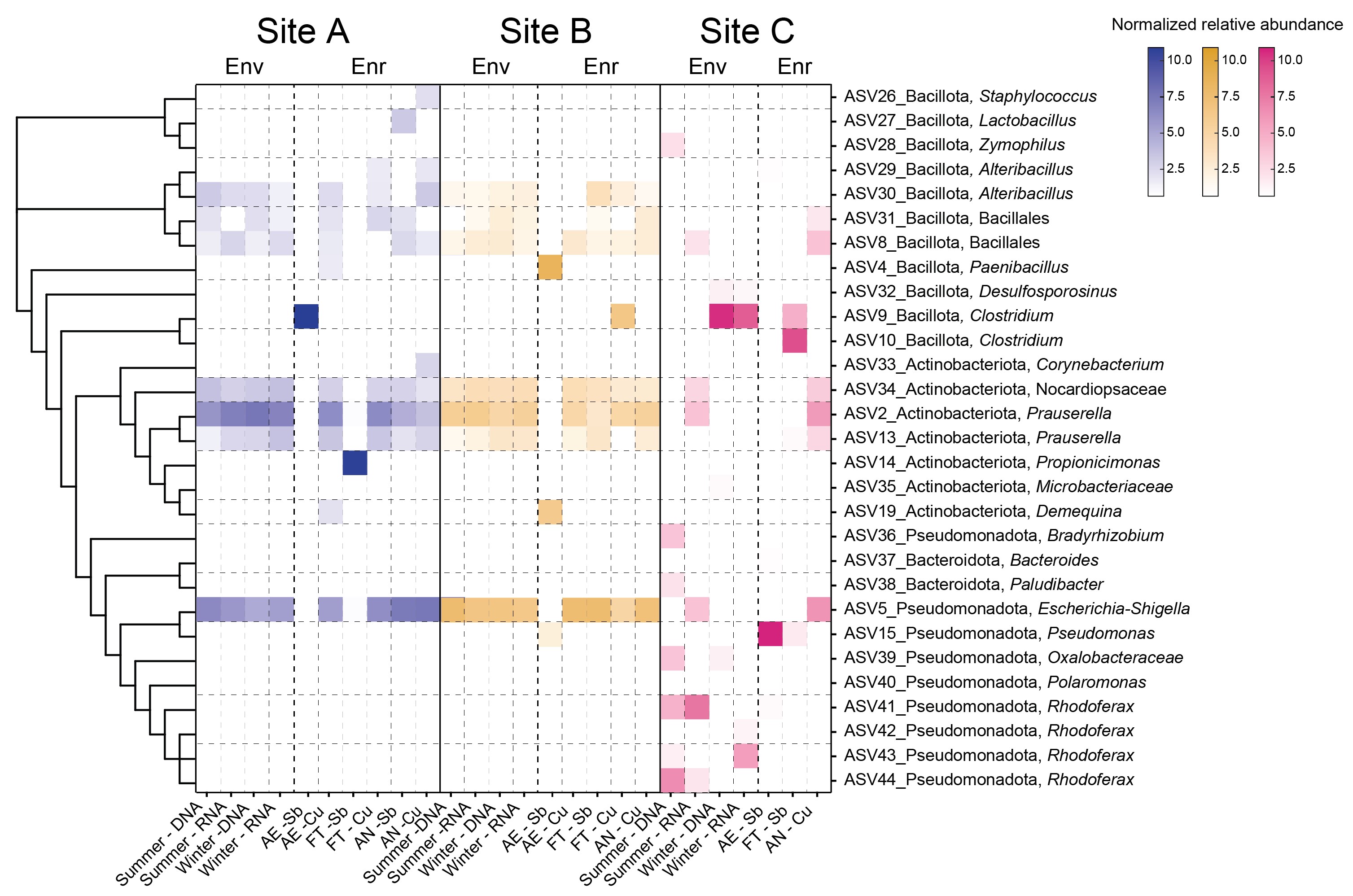

Supplement: SUPPLEMENTARY FIGURE S1 — Pictures of enrichments and isolate cultures. One aerobic Sb-enrichment sample from the third inoculation (A), isolates in anaerobic jars (B), colonies of isolate 68 after a month of inoculation (C) colony of isolate 23 and its halo marked with an arrow (D). [file Data_Sheet_2.zip › Supplementary_Figure_3.jpg]

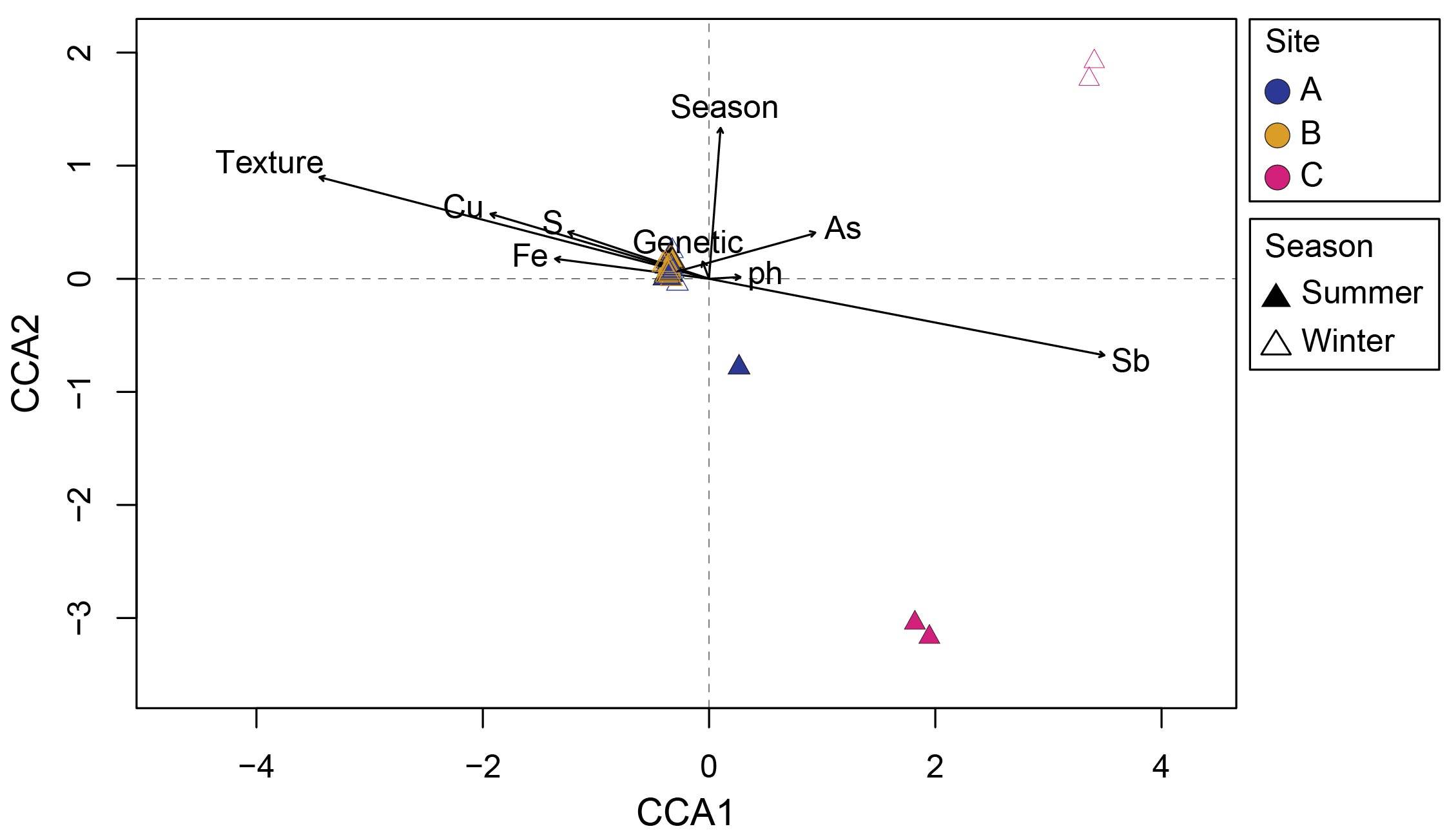

Supplement: SUPPLEMENTARY FIGURE S1 — Pictures of enrichments and isolate cultures. One aerobic Sb-enrichment sample from the third inoculation (A), isolates in anaerobic jars (B), colonies of isolate 68 after a month of inoculation (C) colony of isolate 23 and its halo marked with an arrow (D). [file Data_Sheet_2.zip › Supplementary_Figure_5.jpg]
